# Psychosocial Preparedness for Disasters: A Scoping Review of International Models and Public Health Priorities

**DOI:** 10.1007/s11920-026-01693-1

**Published:** 2026-06-25

**Authors:** Tommaso Barlattani, Alessandra Trianni, Antony Bologna, Edoardo Trebbi, Grazia Terrone, Rodolfo Rossi, Alessandro Rossi, Francesca Pacitti

**Affiliations:** 1 Department of Biotechnological and Applied Clinical Sciences (DISCAB), University of L’Aquila, L’Aquila, Italy; 2 Department of Human Sciences, Università degli Studi Guglielmo Marconi (Unimarconi), Rome, Italy; 3 Department of Public Health and Infectious Diseases, “La Sapienza” University of Rome, Rome, 00100 Italy; 4 Department of Systems Medicine, Tor Vergata University of Rome, Rome, 00133 Italy

**Keywords:** Psychosocial preparedness, Disaster mental health, MHPSS, Disaster risk reduction, Resilience

## Abstract

**Purpose of Review:**

To map international models of psychosocial preparedness for disasters and identify recurrent public mental health priorities for policy, service readiness, and implementation.

**Recent Findings:**

Following PRISMA extension for scoping reviews (PRISMA-ScR) and Joanna Briggs Institute guidance, we searched PubMed (2015–2025) using two complementary strategies and mapped 35 included studies. Eight model families emerged, including community resilience and governance; non-specialist support and psychological first aid (PFA); integrated mental health and psychosocial support (MHPSS) across the disaster cycle; implementation and scale-up models; organizational and health-system preparedness; digital continuity models; monitoring and evaluation frameworks; and behavioral emergency response models. Across model families, preparedness was concentrated mainly on community and non-specialist levels, and emphasized trust, community capacity, task-sharing, workforce readiness, and continuity of care.

**Summary:**

The mapped literature supports psychosocial preparedness as a layered public health function rather than a post-event specialist intervention. Core priorities include pre-event governance, community engagement, supervised non-specialist delivery with referral pathways, workforce protection, digital continuity with clinical safeguards, and minimum monitoring standards. Future work should prioritize implementation-focused research and outcome evaluation across diverse disaster settings.

**Supplementary Information:**

The online version contains supplementary material available at 10.1007/s11920-026-01693-1.

## Introduction

Disasters, pandemics, climate-related emergencies, conflicts, and technological accidents are increasingly recognized as major public mental health challenges rather than purely logistical events. Their effects extend beyond physical injury and infrastructure loss to include prolonged distress, disruption of routines, erosion of social support, and pressure on already fragile health and welfare systems [1–3]. Evidence from Italy after the 2009 L’Aquila earthquake and during the COVID-19 pandemic illustrates both the immediate and the longer-term psychosocial burden of large-scale crises in the general population and in highly exposed groups such as healthcare workers [4–6]. In addition, structural social determinants such as income inequality may shape psychological vulnerability at population level [7]. In this context, preparedness cannot be limited to stockpiles, command chains, and hospital surge plans; it must also include the psychosocial conditions that shape how individuals, communities, and institutions anticipate, absorb, and recover from crises.

Contemporary disaster risk reduction (DRR) frameworks emphasize prevention, preparedness, governance, and recovery across the full disaster cycle [2, 8]. Within this shift, mental health and psychosocial support (MHPSS) has moved from being treated as an optional post-event add-on toward a more integrated, layered model of support. The Inter-Agency Standing Committee (IASC) guidelines conceptualize emergency MHPSS as a four-layer intervention pyramid spanning (1) basic services and safety, (2) community and family supports, (3) focused non-specialized supports, and (4) specialized mental health services [9]. World Health Organization (WHO) and inter-agency guidance reinforce the role of emergencies as opportunities to strengthen systems, embed mental health within public health planning, and build back better after crises [10–12].

Despite this progress, psychosocial preparedness remains unevenly operationalized across sectors. The literature contains frameworks, guidelines, policy papers, and implementation models, but these are dispersed across disaster governance, humanitarian response, community resilience, emergency psychiatry, and digital care, making it difficult to identify consistent operational components and priorities. What remains less clear is how these models converge, which operational components recur most consistently, and how they relate to national systems that already have mature civil protection and community mental health infrastructures.

For the purposes of this review, psychosocial preparedness is defined as the anticipatory set of governance arrangements, community capacities, workforce competencies, referral pathways, and service-continuity mechanisms that enable people, communities, and health systems to anticipate, absorb, and respond to disaster-related psychological distress and social disruption before, during, and after emergencies. Given this heterogeneity, a scoping review was conducted to map and synthesize international models of psychosocial preparedness, identify their recurrent operational components, and derive cross-setting priorities relevant to public mental health and health-system readiness [9–11, 13, 14].

## Materials and Methods

This scoping review followed the PRISMA-ScR and the Joanna Briggs Institute methodological guidance [13, 14]. A scoping approach was chosen due to the conceptual heterogeneity of psychosocial preparedness, which spans empirical studies, reviews, frameworks, policy documents, and implementation models. Accordingly, the aim was to map the available evidence, identify recurrent model families, and derive cross-setting implications, rather than to assess the effectiveness of specific interventions.

A systematic search was conducted in PubMed for studies published between 2015 and 2025, with the final search performed on 1 November 2025. Two complementary search strategies were used to balance sensitivity and specificity: a primary search string combining disaster or emergency contexts with psychosocial, mental health, preparedness, and model-related or guideline terminology; and an extended search incorporating DRR, civil protection, the Sendai Framework, and policy-related terminology. The full search strategies are reported in Appendix A. In parallel, a targeted manual search of institutional documents (WHO, IASC, UNDRR, UNICEF, UNHCR, IOM, IFRC, and Sphere) was conducted to contextualize the scientific evidence within major international MHPSS and DRR frameworks. These documents informed the interpretation but were not included in the PRISMA-ScR study-count denominator.

The systematic review question was framed using a Population-Concept-Context (PCC) approach. The population included human populations, communities, responders, and workforces exposed to disasters, pandemics, humanitarian crises, or public health emergencies. The concept focused on psychosocial preparedness, defined above as an anticipatory public mental health capacity, and on related constructs (e.g., MHPSS, resilience, PFA, readiness, planning, training, referral pathways, service continuity, and capacity building). The context included disasters, public health emergencies, and DRR or civil protection systems.

Eligibility criteria were defined a priori using the PCC approach. Included sources were required to address at least one disaster or emergency context, include a psychosocial, mental health, coping, resilience, or MHPSS component, and incorporate a preparedness dimension (e.g., planning, readiness, training, governance, operational frameworks, guidelines, policies, or implementation models). Articles were limited to English or Italian full-text publications from 2015 to 2025. Sources were excluded if they were outside disaster or DRR contexts, lacked a psychosocial component, focused exclusively on clinical symptoms or treatment without preparedness implications, involved only animal or simulation-based research, or were editorials or opinion pieces without a discernible evidence or policy framework.

**Fig. 1:**
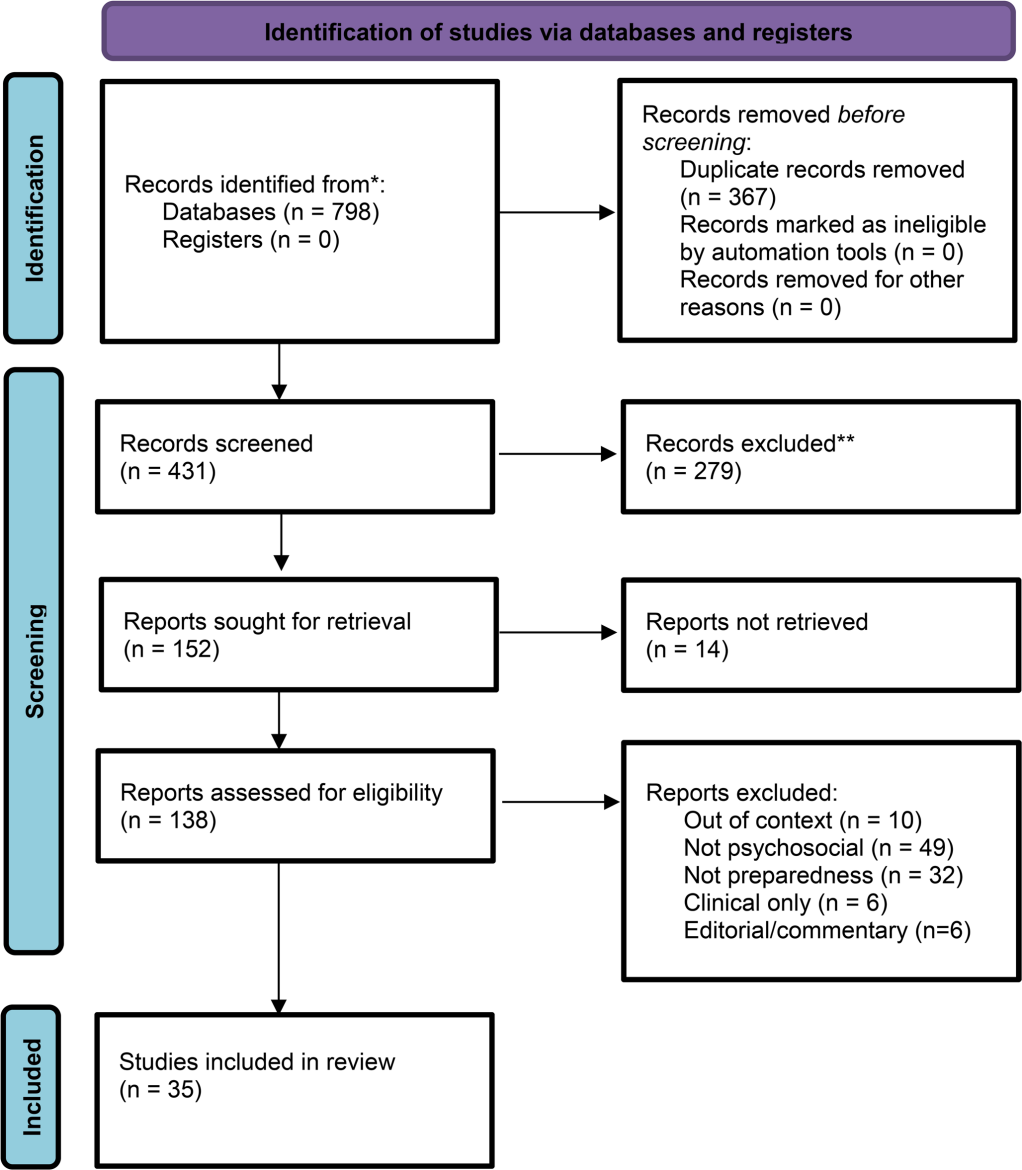
PRISMA-ScR flow diagram of the study selection process

Search results were exported to Zotero^®^ for deduplication and subsequently imported into Rayyan^®^ for screening. Following removal of duplicates, titles and abstracts were screened, followed by full-text assessment of potentially eligible articles. The selection process is summarized in the PRISMA-ScR flow diagram (Fig. 1). The review process followed a predefined methodological framework. Titles, abstracts, and full-text articles were screened independently by two reviewers using predefined criteria. Disagreements at each stage were resolved by a third reviewer.

Data were charted using a structured template capturing study characteristics, context, target population, level of intervention, IASC MHPSS level, model or intervention type, main outcomes or indicators, and implementation status. Charted outputs were summarized in Tables 1, 2 and 3 and Online Resource 1 (Supplementary Tables 1 and 2). The synthesis combined descriptive mapping with qualitative thematic grouping. Studies were grouped into recurrent model families based on their dominant preparedness logic, operational components, and level of action. In line with scoping review methodology, no formal critical appraisal of methodological quality was conducted [13, 14].

## Results

The study selection process is summarized in Fig. 1. The PubMed search produced 798 records of which 367 duplicates were removed. The remaining 431 records underwent title and abstract screening, and 279 were excluded. Full texts were assessed for 152 reports; 14 were not retrieved. Of the 138 full-text articles assessed for eligibility, 103 were excluded for predefined reasons, resulting in 35 included studies in the final synthesis.

The included studies were published between 2017 and 2025, with a marked increase after 2020 and a peak in 2024 (*n* = 10). Most sources were evidence syntheses or reviews (26/35), alongside framework, guideline, or policy-oriented papers (8/35) and one implementation study. The literature was predominantly focused on multi-hazard and pandemic or epidemic contexts, with fewer studies addressing natural disasters, technological events, including nuclear or radiological accidents involving actual or potential ionizing radiation exposure, or behavioral emergencies. Supplementary Table 1 summarizes the main characteristics of the included evidence, while Supplementary Table 2 provides a detailed study-level evidence map of the included sources. Supplementary Tables 1 and 2 are provided in Online Resource 1.

A targeted contextual reading of major international institutional frameworks (e.g., WHO, IASC, UNDRR, IFRC, UNICEF, UNHCR, IOM, and Sphere) informed the interpretation, but was not included in the PRISMA-ScR study-count denominator.

**Table 1:** Summary of psychosocial preparedness model family and representative studies (*n* = 35)

Model family	Key focus	Example studies
Community resilience and governance (*n* = 10)	Trust, participation, risk communication, community infrastructure	Ayub et al. 2023; Bonfanti et al. 2024; Hafez et al. 2024; Houghton et al. 2024; Oktari et al. 2021; Patel et al. 2017; Poland et al. 2021; Pratiti 2023; Roudini et al. 2017; Vandrevala et al. 2024
Non-specialist support and psychological first aid (PFA) (*n* = 4)	Scalable early psychosocial support, task-sharing, referral pathways	Morganstein & Ursano 2020; Orengo-Aguayo et al. 2024; Shah et al. 2020; Sheek-Hussein et al. 2021
Integrated mental health and psychosocial support (MHPSS) across the disaster cycle (*n* = 4)	Coordination, continuity of care, integration across phases	Jacobs et al. 2019; Lee et al. 2019; Ohba et al. 2021; Sandifer & Walker 2018
Implementation and scale-up (*n* = 4)	Adoption, cultural adaptation, sustainability, implementation frameworks	Cohen & Yaeger 2021; Reynolds et al. 2024; Rowe & Nadkarni 2024; Troup et al. 2021
Organizational and health-system preparedness (*n* = 7)	Workforce wellbeing, service continuity, system resilience	Atighechian et al. 2024; Edgar et al. 2022; Herron et al. 2022; Hertelendy et al. 2024; Huang et al. 2025; Kayama et al. 2025; Park et al. 2023
Digital continuity and telemental health (*n* = 4)	Telepsychology, remote care, hybrid service delivery	Alqahtani et al. 2021; Dan et al. 2020; Jaguga & Kwobah 2020; Lyzwinski et al. 2024
Monitoring and evaluation (*n* = 1)	Preparedness indicators, accountability frameworks	Augustinavicius et al. 2018
Behavioral emergency / public safety models (*n* = 1)	Crisis response, de-escalation, public safety interface	Zaiser et al. 2025

Table 1 summarizes the eight recurrent psychosocial preparedness model families identified across the included studies, highlighting their key focus and representative sources. At the family level, the synthesis identified eight recurrent psychosocial preparedness model families, whose core logic, recurrent components, dominant MHPSS levels, and main implications are detailed in Table 2. The largest group conceptualized psychosocial preparedness through community resilience, local governance, trust, and risk communication. These sources emphasized preparedness as a socially distributed capacity built through community engagement, local infrastructures, and trusted messengers rather than through specialist services [15–20, 44–47]. A second group focused on non-specialist support and PFA, conceptualizing preparedness as the capacity to provide early, scalable psychosocial stabilization delivered by trained non-specialists, with referral pathways when needed [21–24].

A third group comprised integrated MHPSS frameworks spanning preparedness, response, recovery, and system reform. These models stressed coordination, continuity of care, and the integration of psychosocial considerations into all phases of emergency management [25–27, 48]. A fourth group addressed implementation science, scale-up, and, to a lesser extent, monitoring. In these sources, preparedness depended on whether models could be adopted, culturally adapted, sustained, and measured across complex settings, often through task-sharing, supervision, information systems, and accountability tools such as theory of change or 4Ws (Who is Where, When, doing What) mapping [28–32].

Organizational and health-system preparedness represented a fifth group, linking psychosocial readiness to workforce wellbeing, service continuity, and operational resilience under pressure [33–39]. A sixth group emphasized digital continuity, including telepsychology, hotlines, mHealth, privacy and consent procedures, and hybrid care models that can maintain access when face-to-face care is disrupted [40–43]. Two smaller groups addressed monitoring and evaluation as a preparedness function [32] and structured responses to behavioral crises at the interface of public safety and mental health [49].

Across model families, psychosocial preparedness was concentrated mainly at levels 1–3 of the IASC MHPSS intervention pyramid [9]. In other words, the literature emphasized basic services and safety, community and family support, and focused non-specialized interventions more often than specialist psychiatry or psychotherapy. Specialist care was generally positioned as a referral or continuity-of-care function rather than the main entry point. Recurrent operational components included community engagement and risk communication, scalable non-specialist support, training and task-sharing, referral and continuity mechanisms, workforce protection, digital continuity, and measurement or accountability structures.

**Table 2:** Main psychosocial preparedness model families

Model family	Core preparedness logic	Recurrent components	Dominant MHPSS levels	Main implication
Community resilience and governance	Preparedness is built through trusted local relationships and social infrastructure.	Risk communication, participation, community leadership, local asset mapping, community engagement.	1–2 (with extensions to 3)	Psychosocial readiness becomes a public mental health and equity function before crisis escalation.
Non-specialist support and psychological first aid (PFA)	Early psychosocial support should be scalable and available beyond specialist services.	Psychological first aid (PFA), train-the-trainer approaches, triage, referral, supervision, just-in-time training.	2–3 (with referral to 4 when needed)	Systems can expand early coverage rapidly without overloading specialist care.
Integrated mental health and psychosocial support (MHPSS) across the disaster cycle	Preparedness, response, recovery, and reform are linked in a single operational logic.	Planning, coordination, continuity of care, recovery governance, build-back-better strategies.	1–3 (with structured links to 4)	Mental health is embedded in DRR rather than added only after the event.
Implementation and scale-up	Preparedness depends on whether models can be adopted, adapted, and sustained.	Task-sharing, cultural adaptation, financing, supervision, information systems, stakeholder engagement.	2–4	Moves the field from aspirational frameworks to sustainable service delivery.
Organizational and health-system preparedness	Psychosocial readiness is part of organizational resilience.	Protocols, exercises, staff support, burnout prevention, crisis communication, continuity planning.	3 (with links to 1–2 and referral pathways)	Protects service continuity and responder functioning under pressure.
Digital continuity	Preparedness includes maintaining access when face-to-face care is disrupted.	Telepsychology, hotlines, mHealth, privacy and consent procedures, digital triage, hybrid care pathways.	3–4	Hybrid care becomes a resilience tool rather than an emergency workaround.
Monitoring and evaluation	Preparedness should be measurable and comparable over time.	Indicators, 4Ws (Who is Where, When, doing What) mapping, theory of change, accountability processes, shared terminology.	Transversal	Enables learning, benchmarking, and policy correction.
Behavioral emergency/public safety models	Preparedness also includes structured responses to acute behavioral crises.	De-escalation, risk assessment, inter-agency coordination, crisis decision support, referral to services.	2–4	Links public safety and mental health within a coherent response pathway.

Synthesizing these model families across settings revealed five recurrent public health priorities (Table 3). First, psychosocial preparedness is most effective when embedded in pre-event governance rather than a late recovery add-on. Second, trust, community engagement, and non-specialist support enable early reach. Third, preparedness depends on effective referral pathways linking community and frontline layers to specialist care. Fourth, workforce wellbeing and service continuity are core components of readiness. Fifth, monitoring and evaluation remain less developed than conceptual and implementation frameworks [15–49].

These priorities were consistent across high-, middle-, and low-resource settings, although implementation varied depending on system capacity and hazard context. Overall, preparedness was best conceptualized as a layered architecture integrating community resources, non-specialist care, governance mechanisms, and access to specialist services, when needed [9–12, 21–43].

**Table 3:** Cross-setting public health priorities for psychosocial disaster preparedness

Domain	Recurrent global insight	Common implementation gap	Cross-setting priority
Governance and coordination	Preparedness is strongest when disaster risk reduction (DRR), health, social care, education, and civil protection have explicit psychosocial roles and pathways.	Psychosocial support is often fragmented and activated mainly after events.	Embed mental health and psychosocial support (MHPSS) in plans, exercises, and cross-sector coordination routines before crises.
Community and non-specialist capacity	Trust, community engagement, psychological first aid (PFA), and task-sharing underpin early reach.	Training, supervision, and referral pathways are uneven.	Build modular training packages linked to referral and supervision systems.
Workforce readiness	Responder and health-worker wellbeing shapes continuity, surge capacity, and quality of care.	Staff mental health protections are frequently reactive.	Standardize peer support, rest/rotation, burnout prevention, and follow-up after high-exposure events.
Digital continuity	Hybrid and remote models can preserve access when services are disrupted.	Privacy, digital inclusion, and clinical governance are inconsistent.	Use digital care with explicit triage protocols, clinical accountability, documentation standards, consent, privacy safeguards, escalation/referral pathways, and equity checks.
Monitoring and learning	Mature models track reach, adoption, continuity, equity, and recovery.	Shared indicators and evaluation frameworks are scarce.	Define minimum indicators and theory-of-change based evaluation for preparedness programs.

## Discussion

This review supports a broad interpretation of psychosocial preparedness as a layered public mental health function rather than a narrow, post-event, specialist-only intervention. Across the included literature, preparedness was not limited to the availability of clinical treatment after disasters. Instead, it was conceptualized as the capacity of communities, organizations, and systems to anticipate psychosocial strain, maintain social functioning, protect vulnerable groups, and preserve access to care through arrangements that begin before crises and extend into recovery.

A central finding was the importance of community and non-specialist layers. In this review, references to “IASC levels 1–3” refer specifically to the four-layer IASC intervention pyramid for MHPSS, rather than to a classification of disasters themselves [9]. Within that framework, level 1 covers basic services and safety delivered in ways that protect dignity and social functioning; level 2 includes community and family supports; level 3 comprises focused, non-specialized supports delivered by trained providers or supervised non-specialists; and level 4 includes specialized mental health services for people with more severe or persistent conditions [9]. Most models identified in this review were concentrated in levels 1–3, while level 4 was generally configured as a referral destination and continuity-of-care function. This distinction is conceptually important because it shows that resilient psychosocial systems depend not only on specialist psychiatry or psychotherapy, but also on trusted local relationships, risk communication, social support, and accessible first-line interventions delivered before needs escalate. From a policy perspective, psychosocial preparedness is therefore closely linked to equity, education networks, primary care, community engagement, and territorial coordination. This orientation aligns with public mental health principles by prioritizing reach, proportionality, and continuity over reactive, specialist-centered approaches.

Implementation remains a critical challenge. While the literature provides many conceptual frameworks, fewer studies address whether models can be effectively adopted, financed, culturally adapted, and sustained. Evidence from implementation-focused studies suggests that preparedness becomes operational only when training, referral pathways, supervision, information systems, and governance mechanisms are established in advance rather than improvised during crises [28–32].

Across settings, elements of psychosocial readiness are often present, but fragmented. Community resources, emergency governance systems, public health services, mental health care, or digital platforms often operate in parallel and are activated at different moments of the disaster cycle. The practical challenge, therefore, lies not only in resource availability, but in coordination across sectors, levels of care, and preparedness phases of the disaster cycle [9–12, 15–20, 28–43].

From a cross-setting public health and systems perspective, five priorities consistently emerged. Psychosocial preparedness should be embedded in pre-event planning and exercises; DRR and MHPSS need shared governance pathways; scalable community and non-specialist interventions require training, supervision, and referral systems; workforce wellbeing must be treated as a core component of operational readiness; and digital and hybrid care models should be supported by clinical governance (i.e., clear triage criteria, clinical accountability, documentation standards, risk-management procedures, supervision, escalation and referral pathways), privacy safeguards, and equity considerations, rather than adopted as emergency workarounds alone [21–49]. Consistent with this point, longitudinal evidence from L’Aquila comparing psychiatric admissions after the 2009 earthquake and during the COVID-19 lockdown suggests that crisis-related service demand may vary by hazard and time horizon, supporting governance arrangements able to monitor access, continuity, and delayed demand [50].

Finally, this review also indicates that preparedness should be monitored using more than activity counts. Mature approaches describe reach, adoption, continuity, equity, and recovery, yet these domains are not consistently measured across settings. Developing minimum indicators and theory-of-change-based evaluations would improve comparability, help decision-makers identify scalable models, and identify scalable and effective preparedness models across hazards, populations, and resource levels [32–49].

## Limitations

Several limitations should be acknowledged. The review was conducted in a single database, PubMed, and limited to English and Italian sources. The included evidence was heterogeneous in design, setting, and level of abstraction. No formal critical appraisal was performed, consistent with scoping review methodology. Fourteen reports could not be retrieved in full text. Contextual institutional documents informed interpretation, but were not included in the PRISMA-ScR study-count denominator. Because this was a scoping review, model-family counts and cross-setting priorities should be interpreted as a descriptive map of the retrieved literature rather than evidence of comparative prevalence, implementation success, or intervention effectiveness.

## Conclusions

Psychosocial disaster preparedness lies at the intersection of disaster governance, public mental health, service design, and community resilience. The mapped international literature supports a layered approach in which psychosocial action begins before emergencies and extends across all phases of the disaster cycle. Across the mapped literature, effective preparedness systems are described as combining community engagement, non-specialist support, implementation planning, workforce protection, digital continuity, and clear pathways to specialist care. For policymakers and service leaders across settings, the most actionable priority is to translate these principles into pre-event plans, training systems, referral pathways, and measurable preparedness indicators.

## Key References

- Bonfanti RC, Oberti B, Ravazzoli E, Rinaldi A, Ruggieri S, Schimmenti A. The role of trust in disaster risk reduction: a critical review. Int J Environ Res Public Health. 2024;21(1):29.
  - ○ This critical review is important because it identifies trust as a foundational condition for disaster risk reduction, public engagement, and effective risk communication. It directly supports this review’s finding that psychosocial preparedness depends on trusted local relationships and credible institutions.
- Hafez S, Ismail SA, Zibwowa Z, Alhamshary N, Elsayed R, Dhaliwal M, et al. Community interventions for pandemic preparedness: a scoping review of pandemic preparedness lessons from HIV, COVID-19, and other public health emergencies of international concern. PLoS Glob Public Health. 2024;4(2):e0002758.
  - ○ This scoping review is important because it synthesizes community interventions across HIV, COVID-19, and other public health emergencies of international concern. It reinforces the argument that preparedness is strengthened when local community capacity is built before crisis escalation.
- Orengo-Aguayo R, Stewart RW, Rodriguez-Sanfiorenzo T del M, Martinez-Gonzalez KG, et al. Implementation of trauma and disaster mental health awareness training in Puerto Rico. npj Ment Health Res. 2024;3:10.
  - ○ This implementation paper is important because it shows how trauma and disaster mental health awareness training can be deployed in a real-world setting. It strengthens the review’s emphasis on workforce readiness, non-specialist training, and operational implementation rather than conceptual planning alone.
- Hertelendy AJ, Howard C, Sorensen C, Ranse J, Eboreime E, Henderson S, et al. Seasons of smoke and fire: preparing health systems for improved performance before, during, and after wildfires. Lancet Planet Health. 2024;8(7):e558-e566.
  - ○ This review is important because it frames wildfire preparedness as a whole-of-health-system performance challenge across pre-event, response, and recovery phases. It supports the conclusion that psychosocial preparedness must include service continuity and workforce protection.
- Lyzwinski LN, McDonald S, Zwicker JD, Tough S. Digital and hybrid pediatric and youth mental health program implementation challenges during the pandemic: literature review with a knowledge translation and theoretical lens analysis. JMIR Ment Health. 2024;11:e52044.
  - ○ This literature review is important because it identifies practical barriers to implementing digital and hybrid mental health services, including access, governance, and sustainability. It underpins the review’s conclusion that digital continuity requires triage, privacy safeguards, and equity checks.

## Supplementary Information

Below is the link to the electronic supplementary material.

Supplementary Material 1

## Data Availability

No datasets were generated or analysed during the current study.

## Appendix A PubMed search strategies (PRISMA-ScR)

Final search date: 1 November 2025

Search strategy 1 (primary search)

(disaster*[tiab] OR emergenc*[tiab] OR “public health emergency“[tiab] OR pandemic*[tiab] OR epidemic*[tiab] OR humanitarian[tiab] OR “mass casualty“[tiab])

AND

(“mental health“[mh] OR “mental health“[tiab] OR psychosocial[tiab] OR “psychosocial support“[tiab] OR “psychological first aid“[tiab] OR mhpss[tiab] OR resilience[tiab] OR “community resilience“[tiab])

AND

(preparedness[tiab] OR “disaster planning“[tiab] OR “emergency preparedness“[tiab] OR readiness[tiab] OR “capacity building“[tiab] OR training[tiab] OR response[tiab])

AND

(model*[tiab] OR framework*[tiab] OR program*[tiab] OR intervention*[tiab] OR guideline*[tiab] OR plan*[tiab] OR protocol*[tiab] OR toolkit*[tiab])

NOT (animals[mh])

Search strategy 2 (extended search)

(disasters[mh] OR disaster*[tiab] OR emergenc*[tiab] OR “public health emergency“[tiab] OR pandemic*[tiab] OR epidemic*[tiab] OR humanitarian[tiab] OR “mass casualty“[tiab] OR “civil protection“[tiab] OR “disaster risk reduction“[tiab] OR DRR[tiab] OR “Sendai Framework“[tiab] OR “surge capacity“[tiab])

AND

(“mental health“[mh] OR “mental health“[tiab] OR psychosocial[tiab] OR “psychosocial support“[tiab] OR “psychological first aid“[tiab] OR PFA[tiab] OR MHPSS[tiab] OR resilience[tiab] OR “community resilience“[tiab])

AND

(preparedness[tiab] OR “emergency preparedness“[tiab] OR “disaster planning“[tiab] OR readiness[tiab] OR “capacity building“[tiab] OR training[tiab] OR response[tiab] OR “surge capacity“[tiab])

AND

(model*[tiab] OR framework*[tiab] OR program*[tiab] OR intervention*[tiab] OR guideline*[tiab] OR polic*[tiab] OR plan*[tiab] OR protocol*[tiab] OR toolkit*[tiab])

NOT (animals[mh])
