# Supplementary material for: Psychosocial Preparedness for Disasters: A Scoping Review of International Models and Public Health Priorities
Source: Curr Psychiatry Rep. 2026 Jun 25;28(1):42. doi: 10.1007/s11920-026-01693-1 (PMC13303767; doi:10.1007/s11920-026-01693-1)
Supplement: Supplementary file 1 — Supplementary Material 1 [file 11920_2026_1693_MOESM1_ESM.docx]

**Psychosocial Preparedness for Disasters: A Scoping Review of International Models and Public Health Priorities**

Tommaso Barlattani ^†^1, 2*****, Alessandra Trianni^†^2, Antony Bologna1, Edoardo Trebbi3, Grazia Terrone^4^, Rodolfo Rossi4, Alessandro Rossi1, Francesca Pacitti1

1 Department of Biotechnological and Applied Clinical Sciences (DISCAB), University of L’Aquila, L’Aquila, Italy

2 Department of Human Sciences, Università degli Studi Guglielmo Marconi (Unimarconi), Rome, Italy

3 Department of Public Health and Infectious Diseases, “La Sapienza” University of Rome, 00100 Rome, Italy

4 Department of Systems Medicine, Tor Vergata University of Rome, 00133 Rome, Italy

*Address correspondence to this author at the Department of Biotechnological and Applied Clinical Sciences (DISCAB), University of L’Aquila, L’Aquila, Italy; E-mail: tommaso.barlattani@graduate.univaq.it

†
These authors contributed equally to the work.

**Online Resource 1. Supplementary Tables 1 and 2**

Supplementary Table 1 Main characteristics of the included evidence map (n = 35)

| Domain | Category | n (%) |
| --- | --- | --- |
| Publication period | 2017-2019 | 6 (17.1%) |
|  | 2020-2021 | 11 (31.4%) |
|  | 2022-2025 | 18 (51.4%) |
| Evidence type | Evidence syntheses/reviews | 26 (74.3%) |
|  | Frameworks, guidelines, or policy papers | 8 (22.9%) |
|  | Implementation study/program | 1 (2.9%) |
| Emergency context | Multi-hazard | 15 (42.9%) |
|  | Pandemic/epidemic | 13 (37.1%) |
|  | Natural disasters | 4 (11.4%) |
|  | Technological or radiological disasters, generic disasters, or public safety emergencies | 3 (8.6%) |
| Model family | Community resilience and governance | 10 (28.6%) |
|  | Non-specialist support and PFA | 4 (11.4%) |
|  | Integrated MHPSS across disaster cycle | 4 (11.4%) |
|  | Implementation and scale-up | 4 (11.4%) |
|  | Organizational and health-system preparedness | 7 (20.0%) |
|  | Digital continuity models | 4 (11.4%) |
|  | Monitoring and evaluation frameworks | 1 (2.9%) |
|  | Behavioral emergency and public safety frameworks | 1 (2.9%) |

**Abbreviations:** MHPSS, Mental Health and Psychosocial Support; PFA, Psychological First Aid.

**Supplementary Table 2** Study-level evidence map of psychosocial preparedness models (n = 35)

| Study | Setting | Evidence Type | Emergency Focus | Core Preparedness Contribution |
| --- | --- | --- | --- | --- |
| **Community resilience and governance (n = 10)**  *Trust, participation, risk communication, and local social infrastructure as preparedness assets.* | | | | |
| Ayub et al., 2023 | Global/ multinational | Narrative review | COVID-19 pandemic | Faith-public health partnerships for trusted RCCE and misinformation management. |
| Bonfanti et al., 2024 | Global/multi-country | Critical review | Natural disaster/ multi-hazard | Trust as a cross-cutting determinant of preparedness, participation, and recovery. |
| Hafez et al., 2024 | Global | Scoping review | Epidemic/ pandemic preparedness | CICICE map of community interventions from informing to empowerment. |
| Houghton et al., 2024 | Latin America & Caribbean | Scoping review | Multi-hazard | Community-driven primary health care resilience and continuity for hazard-prone settings. |
| Oktari et al., 2021 | Multi-country | Delphi study | Multi-hazard | Knowledge-creation elements to strengthen community resilience before disasters. |
| Patel et al., 2017 | Global | Conceptual review | Multi-hazard | Nine core elements of community resilience relevant to preparedness planning. |
| Poland et al., 2021 | Canada | Community framework/ case | Multi-hazard | Connected community approach linking citizens and institutions through local infrastructure. |
| Pratiti, 2023 | Global | Literature review | Multi-hazard | Ecological model integrating social, environmental, and mental health vulnerabilities. |
| Roudini et al., 2017 | Global | Systematic review | Natural disasters | Community mental health preparedness and DRR guidance for natural disasters. |
| Vandrevala et al., 2024 | Global | Systematic review | Multi-hazard | Emergency communication model emphasizing trust, inclusion, and feedback loops. |
| **Early non-specialist support and PFA (n = 4)**  *Scalable stabilization, basic support, and referral beyond specialist care.* | | | | |
| Morganstein & Ursano, 2020 | Global | Narrative review | Ecological/ CBRN hazards | Public mental health framework linking PFA, risk communication, and phased care. |
| Orengo-Aguayo et al., 2024 | Puerto Rico (USA) | Implementation study | Multi-hazard incl. hurricanes, earthquakes, floods, COVID-19 | Scaled MHAT program combining PFA, trauma-informed training, referral, and telepsychiatry. |
| Shah et al., 2020 | Global | Narrative review | COVID-19 pandemic | PFA, RAPID, and just-in-time training as early stabilization tools. |
| Sheek-Hussein et al., 2021 | Global | Narrative review / framework | COVID-19 pandemic | Pandemic psychosocial management with PFA, helplines, infodemic management, and referral. |
| **Integrated MHPSS across the disaster cycle (n = 4)**  *Planning, coordination, continuity, and recovery across all phases.* | | | | |
| Jacobs et al., 2019 | Netherlands | Qualitative meta-syntheses | Multi-hazard | Operational PSS across the disaster cycle: planning, information, registration, and recognition. |
| Lee et al., 2019 | South Korea | Scoping review + Delphi | Disasters | Disaster MHPSS guidelines for pre-event, acute, and longer-term phases. |
| Ohba et al., 2021 | Europe / Japan | Operational framework/ review | Nuclear/ radiological incidents | SHAMISEN recommendations integrating psychosocial support, health surveillance, and risk communication. |
| Sandifer & Walker, 2018 | USA | Narrative review | NaTech/multi-hazard | Stress-reduction framework embedding behavioral health into disaster planning and recovery. |
| **Implementation and scale-up (n = 4)**  *Adoption, cultural adaptation, sustainability, and task-sharing under real-world constraints.* | | | | |
| Cohen & Yaeger, 2021 | LMIC humanitarian settings | Scoping review | Conflict/ humanitarian crises | RE-AIM lens on task-shared refugee MHPSS services and implementation gaps. |
| Reynolds et al., 2024 | Multi-country | Scoping review | Multi-hazard | Implementation science frameworks for migrant and forced-migrant service delivery. |
| Rowe & Nadkarni, 2024 | Global LMICs | Systematic review | Natural disasters | Structural barriers and facilitators for post-disaster MHPSS implementation. |
| Troup et al., 2021 | LMIC humanitarian settings | Systematic review | Humanitarian crises/ displacement | Scale-up conditions for MHPSS, including task-sharing and referral systems. |
| **Organizational and health-system preparedness (n = 7)**  *Workforce wellbeing and service continuity as core preparedness functions.* | | | | |
| Atighechian et al., 2024 | Global | Systematic review | COVID-19 pandemic | Hospital resilience dimensions highlighting staff mental health after COVID-19. |
| Edgar et al., 2022 | Global | Narrative review | Epidemics/ pandemics | Occupational and organizational preparedness recommendations for healthcare workers. |
| Herron et al., 2022 | Pacific Island countries | Health policy paper | COVID-19 pandemic | Eight enablers of emergency care resilience in Pacific Island systems. |
| Hertelendy et al., 2024 | Global | Narrative review | Wildfires / smoke | Health-system preparedness-response-recovery framework for wildfire smoke events. |
| Huang et al., 2025 | Shanghai, China | Operational framework | Urban multi-hazard | Resilience pathways for public health rapid response teams. |
| Kayama et al., 2025 | Japan | Policy/ framework paper | COVID-19 pandemic | Nursing capacity-development framework for the next pandemic. |
| Park et al., 2023 | South Korea | Systematic review/ guideline | COVID-19 pandemic | Burnout prevention and workforce mental health within infection-control planning. |
| **Digital continuity and telemental health (n = 4)**  *Remote and hybrid pathways to preserve access when services are disrupted.* | | | | |
| Alqahtani et al., 2021 | Saudi Arabia | Guideline/ protocol | COVID-19 pandemic | Telepsychology guideline covering consent, privacy, triage, documentation, and referral. |
| Dan et al., 2020 | China | Policy scoping review | COVID-19 pandemic | Chinese policy response using hotlines, online platforms, and rapid workforce deployment. |
| Jaguga & Kwobah, 2020 | Kenya/sub-Saharan Africa | Policy review | COVID-19 pandemic | MHPAF framework combining governance, PFA training, tele-support, and surveillance. |
| Lyzwinski et al., 2024 | Canada + international evidence | Literature review | COVID-19 pandemic | Digital and hybrid youth mental health programs and implementation requirements. |
| **Monitoring and evaluation (n = 1)**  *Preparedness as a measurable and accountable function.* | | | | |
| Augustinavicius et al., 2018 | Multi-country humanitarian settings | Scoping review/ document analysis | Multi-hazard humanitarian settings | M&E frameworks (logframes, theory of change, 4Ws) for standardized humanitarian MHPSS. |
| **Behavioral emergency / public safety interface (n = 1)**  *Structured crisis decision support at the interface of public safety and mental health.* | | | | |
| Zaiser et al., 2025 | International | Conceptual framework | Behavioral/ public-safety emergencies | iBEAR framework for structured behavioral emergency response and de-escalation. |

**Abbreviations:** DRR, Disaster Risk Reduction; iBEAR, **Integrated Behavioral Emergency Assessment and Response; MHPAF, Mental Health Preparedness and Action Framework;** MHPSS, Mental Health and Psychosocial Support; PFA, Psychological First Aid; RCCE, Risk Communication and Community Engagement; MHAT, Mental Health Awareness Training; NaTech, Natural Hazard Triggering Technological Disasters.
